# Interhemispheric Control of Unilateral Movement

**DOI:** 10.1155/2012/627816

**Published:** 2012-12-06

**Authors:** Vincent Beaulé, Sara Tremblay, Hugo Théoret

**Affiliations:** ^1^Department of Psychology, University of Montreal, Montreal, QC, Canada H3C 3J7; ^2^Research Center, Sainte-Justine Hospital, University of Montreal, Montreal, QC, Canada H3T 1C5

## Abstract

To perform strictly unilateral movements, the brain relies on a large cortical and subcortical network. This network enables healthy adults to perform complex unimanual motor tasks without the activation of contralateral muscles. However, mirror movements (involuntary movements in ipsilateral muscles that can accompany intended movement) can be seen in healthy individuals if a task is complex or fatiguing, in childhood, and with increasing age. Lateralization of movement depends on complex interhemispheric communication between cortical (i.e., dorsal premotor cortex, supplementary motor area) and subcortical (i.e., basal ganglia) areas, probably coursing through the corpus callosum (CC). Here, we will focus on transcallosal interhemispheric inhibition (IHI), which facilitates complex unilateral movements and appears to play an important role in handedness, pathological conditions such as Parkinson's disease, and stroke recovery.

## 1. Introduction

Humans have a natural tendency towards symmetrical contraction of homologous muscles (also called voluntary mirror movements), which are known to require less cortical activation than alternated bimanual movements or unilateral movements [1, 2]. For example, it has been shown that if bimanual movements are executed with the upperlimbs, there is a strong tendency towards synchronization of motor patterns [3]. This is why the execution of strictly unilateral motor movement requires complex interhemispheric interactions between a wide range of cortical areas. These interactions are needed to restrict motor output to the contralateral primary motor cortex (M1) that controls the intended hand movement, which belongs to the “nonmirroring” transformation network [4]. Experimental and clinical data suggest a relevant role of the corpus callosum (CC) in this network. For example, children, who have an immature CC, have a higher incidence of mirror movements (MM), as do some patients with agenesis of the CC [5]. This network enables healthy adults to perform strictly unilateral tasks, although some subtle MM can be observed in the unused hand if the task is complex or fatiguing [6].

Any dysfunction in the complex network that underlies unilateral movement, which relies in part on inhibitory interhemispheric interactions, can contribute to the presence of MM. In the present paper, we will review specific aspects of the nonmirroring transformation network to higlight its role in lateralization of voluntary movements. Current data regarding physiological mirroring seen in healthy adults and the role of IHI in the lateralization of movements will also be discussed. Finally, the neuroanatomical substrates of the nonmirroring network and the effects of aging on the reappearance of MM will be presented. 

## 2. Mirror Movements

### 2.1. MM in Children

MM are movements that are observed in the contralateral hand that are the mirror reversals of the intended movement of the active hand. MM observed in children are explained by an undermyelinated nervous system [5, 7] that does not permit the interhemispheric communication necessary for the restriction of motor output to the M1 contralateral to the intended movement. The corpus callosum (CC) is the biggest white matter bundle of the brain and its function is to connect both hemispheres [8, 9], and it appears that its incomplete myelinisation could partly explain MM in healthy children [5]. However, it is important to note that immaturity of other parts of the distributed “nonmirroring” network may also play an important role in MM seen in children. MM are seen in healthy children and decrease with increasing age until the age of 10 years [5, 10, 11]. There seems to be a substantial decrease in MM, particularly from 5 to 8 years of age, which has led some authors to suggest that it may serve as a developmental cue [12, 13]. The overt MM seen in healthy children disappear with maturation of the central nervous system and myelinisation of the CC [5, 14, 15], and there is a significant relationship between chronological age and size of the CC [16]. As the myelinisation of the CC occurs, there is a concomitant increase in IHI between the two motor cortices, which could also be used as a marker of motor development [17]. Therefore, the ability to execute unilateral motor tasks seems to rely on the correct maturation of this transcallosal inhibitory system since impaired inhibition is associated with MM [7, 18]. Notwithstanding these observations, MM have been only sporadically reported in patients with agenesis of the CC, whereas most acallosal patients display no overt MM, suggesting a relative role played by the CC in MM. Because of the reduced IHI seen in children, the ipsilateral M1 controlling the mirror hand has a higher level of excitation if the voluntary hand is active. Consequently, bilateral cortical activity can be recorded in healthy children when performing a unilateral task [5], which enhances the probability of MM. In healthy adults, transcallosal IHI presumably suppresses activity in the ipsilateral M1 resulting in strictly unilateral movements. MM seen in healthy children thus seem to be the result of the bilateral activity of both M1 when performing a unilateral task. These observations provide strong evidence that a mature central nervous system capable of transcallosal IHI is a key factor in controlling unwanted MM when performing a unimanual task. 

### 2.2. The Role of Interhemispheric Inhibition in Unilateral Movements

To perform strictly unilateral movements there is a “nonmirroring” process that restricts the motor output in the contralateral hemisphere and suppresses motor activation of the mirror hand [19, 20]. When an individual is preparing to execute a finger movement, it is followed by a temporary inhibition of the homologous M1 controlling the mirror finger in the passive hand [19]. TMS-induced MEPs are progressively facilitated in the 80–120 ms preceding EMG onset in healthy adults [21–23]. This shift in facilitation of the contralateral M1 and inhibition of the ipsilateral M1 following intended movements could be linked to interhemispheric interactions of the two M1. If a participant is asked to prepare a hand movement in a reaction time paradigm and a conditioning TMS pulse is applied over the ipsilateral M1 followed by a test pulse over the contralateral M1, IHI will be stronger immediately after the go signal. But as the voluntary movement onset nears, IHI is released, leading to increased excitability [24–26]. By comparison, if the conditioning pulse is delivered to the contralateral M1, which will then inhibit the ipsilateral M1, the inhibition remains deep from the beginning through the end of movement preparation [24–26].

It has been suggested that the ability to perform unilateral finger movements without MM depends on the appropriate modulation of IHI between the contralateral and ipsilateral M1 that occurs during movement preparation [19, 25, 27]. This hypothesis, that the transcallosal increase in IHI originating from the active M1 to the mirror M1 is responsible for the inhibition of undesired MM, was tested during a bimanual motor task by Hübers and colleagues [28]. In that study, participants had to maintain a tonic isometric contraction of the mirror hand while the active hand was executing short-duration contractions. It was found that IHI was inversely correlated with motor overflow in the mirror hand. That is, the more the M1 contralateral to the active hand executing short contractions (M1-active) was able to inhibit the ipsilateral M1 (M1-mirror), the less mirror activity was seen in the mirror hand performing a tonic isometric contraction. This phenomenon was further tested using low-frequency rTMS to interfere with the M1-active to determine whether releasing inhibition from the M1-active would enhance mirror activity in the hand maintaining the tonic contraction. In line with the hypothesis, less IHI from the active M1 to the mirror M1 was found, resulting in increased mirroring [28]. This further suggests that the active M1 is partly responsible for the inhibition of MM through IHI to the mirror M1. Similarly, Kobayashi and colleagues [29] reported that low-frequency rTMS over M1 resulted in enhanced motor performance (finger-tapping task) of the ipsilateral hand. Notably, the increase in performance was associated with increased excitability of the unstimulated M1, which was possibly obtained by suppressing inhibition from the stimulated M1 to the contralateral M1. It was later shown that the same protocol could improve the learning of a simple motor task in the ipsilateral hand while disturbing learning in the contralateral hand [30]. These two studies suggest that when a unilateral hand movement is executed, activation of the active M1 has an influence on the contralateral M1, acting as a “brake” that, when withdrawn, can disinhibit the contralateral M1 and lead to behavioral improvement [31]. Therefore, it seems that IHI modulation is crucial for restricting the motor output to the contralateral M1 and inhibiting the mirror M1 for an accurate, strictly unilateral, movement of the hand. 

Additional evidence for the presence of mutual inhibition between motor cortices comes from stroke patients, where IHI towards the affected M1, which controls the paretic hand, is increased [24]. Moreover, it appears that stronger IHI towards the affected hemisphere is negatively correlated with motor function recovery, suggesting a direct relationship between increased IHI from the intact hemisphere to the lesioned hemisphere and poor recuperation of motor function in chronic stroke patients [24]. Following on this, it is not surprising applying low-frequency rTMS over the nonaffected M1 to reduce its excitability can improve motor function in the paretic hand of stroke patients through a mechanism by which transcallosal inhibition from the nonaffected hemisphere is released, leading to increased excitability and function of the affected M1 [32]. 

IHI between the motor cortices can also be tested by the brief interruption or attenuation of voluntary EMG activity produced by focal single-pulse TMS of the ipsilateral M1, the so-called ipsilateral silent period (iSP) [33]. Similarly to IHI [34], the iSP is mediated by a transcallosal pathway [35, 36], but the two measures appear to rely on different neural substrates [37]. There is evidence that the iSP reflects a key phenomenon that helps restrict motor output in the contralateral M1. Indeed, it seems that activation of the M1 performing a voluntary movement with the contralateral hand increases interhemispheric inhibition as measured by the iSP. This evidence points to a pivotal role for the mechanism underlying the iSP in suppressing unwanted MM and controlling unilateral movement [33].

Numerous factors have been reported to modulate interhemispheric interactions. For example, in a force generation task, it was shown that when a participant is maintaining a contraction at 70% maximal force, IHI has a disinhibitory effect on the M1 ipsilateral to the voluntary contraction, as reflected by reduced short intracortical inhibition (SICI). This suggests a change in IHI depending on task features [38]. Along the same line, it has been shown that IHI differs depending on which arm muscle is tested. Indeed, IHI from different arm representations does not strictly follow a “proximal-to-distal” gradient but instead may depend on the role that each muscle plays in functional movement synergies [39]. Finally, it has also been reported that training can modulate IHI. This was pointed out in a study testing professional musicians, who require enhanced coordination. It was found that IHI is lower in musicians as compared to controls, suggesting a modulatory effect of training on IHI [40]. 

### 2.3. Physiological Mirroring in Healthy Adults

It is known that the amount of mirror EMG activity seen in healthy subjects increases if the task is demanding, if fatigue is induced, if there are cognitive distractions or decreased attentional capacities, and if age increases [41–45]. A protocol has been developed to probe physiological mirroring in healthy adults, following the observation that facilitation of the motor response can be achieved by simultaneous contraction of ipsilateral and contralateral hand muscles [46, 47]. This leads Mayston and collaborators [5] to report that involuntary mirror EMG activity of the right first dorsal interosseous (FDI) muscle can be induced in healthy adults if they maintain a background isometric muscle contraction with the mirror FDI (right) while performing intended unilateral brief phasic contractions with the left homologous muscle, resulting in motor overflow to the right hand. This protocol has been used in numerous studies where it has been repeatedly shown that mirror activity can occur in healthy participants [5, 48, 49], which is assumed to result from the transfer of activation from the task-M1 to the mirror-M1 through the CC [48].

Since physiological mirroring in healthy individuals cannot be explained by an ipsilateral projection originating from the M1 contralateral to the intended movement, alternate mechanisms must be proposed. There is growing evidence suggesting that physiological mirroring depends on the activation of the ipsilateral M1, which normally has a crossed CS tract connecting to the mirror hand [50–52]. This transfer of activation is thought to occur through a transcallosal pathway [1, 20, 34]. Therefore, the CC seems to play an important role in restricting motor overflow since, through callosal fibers, each M1 can have an interhemispheric influence over the other. This influence can either be a direct excitatory effect or an indirect inhibitory effect by excitating inhibitory interneurons [44, 53]. Evidence for a transcallosal role in IHI comes in part from studies showing that patients with agenesis of the CC display no IHI [35, 54] and that children have no IHI and an immature CC [5]. With this in mind, some authors have suggested that an intact and fully myelinated CC is necessary for effective IHI, which is also important to suppress activity in the contralateral M1 [28]. 

The relationship between a functional CC and IHI was investigated in a study that combined TMS and diffusion tensor imaging (DTI). A direct correlation was found between fractional anisotropy (FA) of the CC, which represents the coherence of diffusion of water molecules along the WM tract, and the strength of IHI evaluated by Ferbert's paired-pulse TMS protocol [55]. Other studies have investigated the link between IHI and measures of FA in the CC in healthy humans [56] and patients with WM dysfunction [57] and suggest that WM tract integrity can be used as a predictor of IHI in healthy and diseased individuals [55, 58]. These results confirm that proper myelination of the CC is important since it enables rapid conduction of nerve impulses and, at the same time, isolates axons to prevent unwanted interference to enhance quality of interhemispheric communication coursing through the CC [5].

The CC appears to play an important role in the control of unilateral movements and in preventing mirroring by facilitating interactions to keep motor outputs contralateral to intended movements [35, 53]. This is in line with studies in monkeys, where species that do not display bimanual skills do not possess direct transcallosal M1-M1 connections [59], whereas in macaques, in whom M1-M1 connections are found, skilled coordination abilities are seen [60]. Similarly, in humans, studies with patients with an abnormal CC have shown that it is crucial for fast and complex unilateral and bilateral coordination, as well as for the ability to learn new bimanual skills [61–63]. Other evidence for a crucial role of the CC as part of the nonmirroring transformations network comes from patients with schizophrenia. There is growing evidence that the development of the CC is abnormal in schizophrenia, leading to impaired transcallosal connectivity of the two hemispheres [64]. This was confirmed using DTI and MRI, in which structural abnormalities and reduced volume of the CC were observed [65] for first-episode patients as well as high-risk individuals [66]. It was suggested that CC abnormalities could result in neurological soft signs (NSSs) [67]. NSSs have a high prevalence in schizophrenia, with 50–65% of patients being affected [68]. Individuals with schizophrenia display higher levels of motor overflow in the nonactive hand compared to controls [67]. The higher incidence of mirroring activity in the nonactive hand of schizophrenia patients was later associated with deficient intracortical inhibition originating from the M1 ipsilateral to the active hand [69], which in turn could be associated with reduced IHI between the active and nonactive M1. With low IHI, each M1 shows a higher degree of excitation expressed by lower intracortical inhibition, thus enhancing the possibility that overflow occurs in the nonactive hand [29, 69].

### 2.4. MM in Adults

If MM persist after the age of 10, they are considered pathological. There are different genetic aetiologies that can explain the persistent presence of MM. They can be seen in adults that do not have other motor abnormalities and are then called congenital mirror movements (CMM) [54, 70–73]. They can also be found in genetic syndromes like Kallmann's syndrome [74–76], Klippel-Feil syndrome [77, 78], congenital hemiparesis [79, 80], and schizophrenia [67, 81, 82]. MM can also reappear later in life in acquired conditions like Parkinson's disease (PD) [49, 83], stroke [84, 85], and can be present in normal aging [43, 44, 86].

The neurophysiological hallmark of CMM is the presence of an ipsilateral, fast-conducting corticospinal tract connecting the contralateral M1 to the ipsilateral hand. Evidence for this aberrant ipsilateral connection comes in part from transcranial magnetic stimulation (TMS) studies where it has been repeatedly shown that in individuals with CMM, the ipsilateral motor evoked potential (MEP) elicited, while at rest by a single TMS pulse over M1 has the exact same latency as the contralateral MEP [72]. This rules out the possibility that the involuntary mirror electromyographic (EMG) response is the result of a transcallosal transfer of excitability from the contralateral M1 to the ipsilateral M1 since such a transfer is expected to take about 8-9 ms [87]. Along the same lines, Lepage and collaborators [54] reported the case of a patient with agenesis of the corpus callosum (CC) showing ipsilateral and contralateral MEPs of the same latency.

Some evidence suggests that a gene, the deleted in colorectal carcinoma (DCC), may be responsible for CMM [88–90]. The DCC gene stands for deleted in colorectal cancer and is a receptor for netrin-1, which is a protein necessary for axon guidance across the body's midline [89–91]. It seems that there is a genetic heterogeneity that causes CMM since three different mutations on the DCC gene have been reported to cause CMM in three different families [90]. The role of the DCC gene has been confirmed by the fact that “knocking out” the DCC gene or the ephrin gene in a mouse results in movements being synchronized in a mirror-like fashion [91, 92]. This suggests a possible misdirected ipsilateral corticospinal projection occurring when the CS tract crosses the midline, possibly explaining the presence of MM in this population [89]. However, in several other familial cases of CMM, no DCC mutations have been identified, which led to the discovery of a novel gene responsible for CMM [93]. It was found that a mutation on the RAD51 gene could also lead to CMM. The RAD51 gene is mostly present in the mouse cortex at a developmental stage critical for the correct establishment of the corticospinal tract (CST) [93]. These findings strongly suggest that CMM reported in otherwise healthy adults are the result of specific mutations that affect either the DCC or RAD51 genes culminating in an aberrant ipsilateral CST. 

### 2.5. MM in Pathological Conditions

MM are also seen in specific conditions. Kallmann's syndrome (KS) is mainly characterized by hypogonadotrophic, hypogonadism, and anosmia [75]. However, only the X-linked form of KS is associated with MM [74]. Mayston and collaborators [75] suggested an abnormally developed ipsilateral tract as an explanation for MM in KS, as MM exhibited by KS patients had the exact same latency as the contralateral voluntary response. Also, it was reported that mirror responses decreased in size at the same time as the contralateral response if the TMS coil was moved away from the maximum response region, suggesting that the ipsilateral and contralateral corticospinal (CS) axons projecting to both hands are connected to the same M1 in both hemispheres. The pathological ipsilateral tract in XKS is suggested to innervate bilateral motoneurons of the distal upper limb muscles with a variable size effect as measured by MM [76]. MM are also found in Klippel-Feil syndrome (KFS), which is characterized by a short neck, impaired cervical mobility, and low airline [77] and is commonly associated with MM [78]. In these patients, the MM are mainly observed in the distal upper limb muscle [77]. An autopsy of a deceased patient with KFS revealed the absence of pyramidal decussation of the CS tract [94]. The bilateral motor responses exhibited in KFS patients show comparable properties to contralateral responses seen in healthy controls [77]. In patients with MM in KFS [77] and XKS [75], if they are performing unimanual voluntary movements, it is possible to observe a short duration central peak in the cross-correlograms obtained from multiunit EMG activity recorded simultaneously from both homologous muscles. This activity contrasts with control subjects who only display a contralateral response, thus adding weight to a possible abnormal corticospinal branching of their motor cortex projection. MM may also occur in patients with severe congenital hemiparesis [79, 80]. In this pathological condition, the unaffected motor cortex has abnormal ipsilateral corticospinal fibers branching to the paretic hand, thus resulting in MM. Interestingly, these patients are, to some extent, capable of lateralized motor activity [79]. This was reported in an experiment where mirror hand activity was recorded with EMG, showing that it was less activated than the hand performing the intended contraction. It was thus suggested that a reorganization of the CST in these patients results in separate pathways connecting the unaffected motor cortex to both hands. It was shown that an intended contraction of the paretic hand is followed by an inhibition of the crossed CST to the good hand, as seen with reduced MEPs in the mirror hand compared with a rest condition [79]. This suggests that in patients with hemiparesis, the unaffected motor cortex is able to inhibit homologous motor representations [79].

### 2.6. The Case of Right Handedness

There is growing evidence suggesting that manual preference in the use of one hand could be explained by an asymmetry in IHI [26]. For example, IHI from the dominant M1 towards the nondominant M1 was compared with IHI from the nondominant M1 towards the dominant M1, where it was found that the former was deeper [95]. This was also shown by Duque and colleagues [26], who reported that modulation of IHI with movements of the right and left hands in right-handed healthy subjects was asymmetrical. In the preparation of movement, the balance in IHI was profound in both hemispheres, which could help restrict MM during unilateral motor tasks. However, as movement onset approached, an asymmetry began to appear, revealing increased disinhibition of the contralateral M1 during right hand movement compared with left hand movement. The shift in IHI leading to higher excitation was only seen when the right hand was performing the task, whereas IHI towards the nondominant M1 remained deep. In the nondominant M1, when the left hand was performing the task, there was an almost constant IHI balance towards each hemisphere [26]. This asymmetrical modulation of IHI with regards to right and left hands movement could play an important role in fine motor coordination of the dominant hand. The release of inhibition from the nondominant hemisphere to the dominant hemisphere executing a task with the contralateral hand leads to enhanced excitation of the dominant hemisphere while at the same time maintaining a deep inhibition of the nondominant mirror hemisphere, which restrains the occurrence of MM [96]. This important excitatory gain could allow more refined movements by the dominant hand through effective intracortical excitatory connections of the dominant hemisphere with better control over antagonistic and irrelevant representations [97].

It has been suggested that in order to counteract higher IHI towards the nondominant M1 controlling the left hand in right-handed subjects, the nondominant M1 has to recruit more corticospinal neurons to accomplish comparable performance to that of the right hand [98]. This has led to the hypothesis that the increased mobilization of corticospinal neurons required by the nondominant hand could express itself in the form of interhemispheric facilitation towards the ipsilateral, dominant M1 [26]. It could also be an adaptive mechanism aimed at counteracting the higher levels of inhibition targeting the right hemisphere. Since the right M1 inhibits the left M1 at lesser levels, through IHI, when the right M1 is active in a task, the left M1, which is not fully inhibited, could maintain slight IHI towards the right M1 forcing the right M1 to recruit more CS neurons to perform as well as the right hand. There is evidence for this asymmetry, as it was found that the ipsilateral dominant cortex is more active during left hand movement than the ipsilateral nondominant cortex during right hand movements [6, 19]. It is thus possible that the left M1 activity that is seen during left hand movement results in persistent IHI towards the nondominant active M1, leading to poorer performance with the nondominant hand [26]. Along the same lines, increased MM in the right hand of right-handed subjects might be a consequence of the left M1 contribution during hand movements performed with the nondominant hand [99]. Taken together, these data may partly explain why the protocol used by Mayston and collaborators [5] can induce mirror movements in healthy participants more easily when the right hand maintains the tonic, isometric contraction, while the left hand performs brief movements. The tonic contraction of the dominant right hand keeps the left M1 activated, which then leads to greater IHI towards the right M1. This higher inhibition in the right M1 could then result in even lower IHI towards the dominant M1, which has greater excitability, as shown by reduced intracortical inhibition [100]. This higher excitation level in the dominant M1 makes it more vulnerable to the excitation that is produced by bimanual movement, which has to be inhibited [35]. However, since the dominant M1 is overexcited, it results in slight motor overflow. This is more easily achieved with the right hand because if the left hand is tonically contracted, the right M1 produces less IHI towards the left M1, which is then able to restrict motor overflow, without expanding it to the contralateral hemisphere. At the same time, IHI of the dominant M1 is also stronger, lowering the activation of the contralateral M1, thus producing lower motor overflow in the nondominant left hand [48]. However, it should be noted that MM in the nondominant hand using the Mayston protocol have also been reported [48]. It should be mentioned that IHI from the dominant to the nondominant M1 is but one of the mechanisms that have been proposed to explain hand dominance. For example, there is evidence that enhanced efficiency of motor neurone synchronization may be present in the arm preferentially used by an individual [101]. It has also been suggested that that a release of inhibitory input to the contralateral M1 from a more strongly activated right M1 may facilitate better *bimanual* coordination [102].

## 3. Neuroanatomical Substrates

To perform unilateral movements, the brain relies on a largely distributed network of motor cortical and subcortical areas, which is called the nonmirroring network. The understanding of this network and the mechanism involved in restricting motor output to the contralateral muscle, which requires the transformation of a default bilateral MM to a lateralized unilateral movement, is starting to emerge. Data from healthy humans, patients, and lesioned monkeys support the view that this network relies on the supplementary motor area (SMA) [103], the dorsal premotor cortex (dPMC) [1], the ipsilteral M1 [26, 28], and the basal ganglia [83].

### 3.1. Dorsal Premotor Cortex

Studies using positron emission tomography have shown that right dorsal premotor cortex activation is more important during out-of-phase bimanual movements compared to in-phase movements, also known as voluntary MM [104]. This points to a role for the dPMC in the nonmirroring process since it is recruited more prominently when asymmetrical movements are required compared to voluntary mirroring. The functional importance of the dPMC in the nonmirroring process was confirmed by an rTMS study where stimulation was applied over the right dPMC of healthy participants while performing a unilateral contraction of the left hand. It was shown that disruption of the right dPMC increased excitability of the CS projections from the left M1 to the right mirror hand. This was seen only if the left hand was performing a voluntary contraction [1]. This suggests that the right dPMC plays a role in the nonmirroring network responsible to the restriction of motor output in the right M1 when the left hand is performing a unilateral manual task. This idea was further supported by Giovannelli and colleagues [49], where they showed that low-frequency rTMS of the right dPMC enhances physiological mirroring in healthy adults. It should be noted that in both of these studies, stimulation of dPMC resulted in no overt MM, although slight motor overflow was present in the mirror hand. This suggests that the dPMC is part of a network of areas underlying nonmirroring transformations [1] that contribute in restricting the motor output to the hemisphere contralateral to the intended movement [1, 49].

### 3.2. Supplementary Motor Area

There is evidence suggesting a role for the supplementary motor area (SMA) in the nonmirroring cortical network since unilateral ablation of the SMA in monkeys produces long-lasting decreases in bimanual coordination, with greater effect if the lesion is located in SMA contralateral to the nondominant hand [105]. Additional lesion evidence comes from the report of a man who suffered an infarct to the right SMA and in which mirror movements were seen when writing and performing bimanual coordination tasks [103]. This suggested that the SMA was part of the nonmirroring transformation of motor programs which originated in the left hemisphere prior to execution by the right M1 of left hand movement [103]. Similarly impaired motor control was seen in three patients with unilateral ablation of the SMA to help control epilepsy, in which alternating movements were impaired in the hand needing reciprocal coordination [106]. Neuroimaging in healthy humans has also revealed greater activation of the SMA when bilateral, asymmetric movements are performed compared with symmetric movements, similarly to what is observed in dPMC [2, 104]. SMA involvement in nonmirroring transformations can also be seen anatomically since it projects bilaterally to M1 via the CC and reaches the PMC and the contralateral SMA [107]. In fact, M1 receives its major ipsilateral projection from the SMA [108]. The role of the SMA in motor control seems crucial since disturbances in bimanual coordination that include MM may be present in patients with SMA damage [103].

The idea that the nonmirroring program of motor control relies on a large neural network involving the dPMC and SMA is supported by studies using scalp movement-related cortical potentials (MRCP). It seems that both unilateral and bilateral voluntary movements are preceded by a premovement EEG potential called the Bereitschaftspotential (BP), which is a slow negativity, that is, bilaterally distributed over extensive areas of the scalp and that occurs approximatively two seconds before movement onset [109, 110]. With regards to hand movements, the main source of this “early” BP is believed be located in the bilateral SMA and lateral precentral gyrus [110], although some studies have reported higher amplitudes over the contralateral SMA [111]. This premovement activity suggests a role for the SMA in the preparation of upcoming movement and its bilateral presence, in addition to its connection to ipsilateral and contralateral M1 [108], which makes the SMA a perfect candidate for an integrative role in coordinating bimanual movements [112]. Following the early BP, there is an increase in its gradient approximatively 400 ms before movement onset, which exhibits a markedly different scalp distribution and is called Negative Slope (NS0) [113]. The NS0 originates in M1 and PMC and shows precise somatotopy [110, 111], and if it is bilaterally distributed during unilateral hand movements, rather than being predominantly contralateral, bilateral activation of M1 is present and may result in MM, probably through the lack of transcallosal inhibition [110]. Following contralateral NS0 is the motor potential (MP), which peaks concurrently with movement onset. The MP is localized in a restricted area of the contralateral scalp and is thought to reflect the activity of pyramidal tract neurons taking place in the contralateral M1 [110]. These findings are in agreement with the SMA playing a major role in the preparation of movement, since it is activated early during motor preparation and is bilaterally distributed. This bilateral activation is followed by restricted contralateral activations in PMC and M1, which in turn will give rise to strictly unilateral movements. 

### 3.3. Basal Ganglia

It has also been suggested that the basal ganglia could play a substantial role in sequential movements, in the timing movements, and in selecting the muscles required for a motor task, as well as for the execution of overlearned motor programs [114]. With this in mind, it is not surprising that the SMA receives strong indirect projections from the basal ganglia (GPi) via the thalamus [115]. In PD, evidence points out to impaired basal ganglia function through depleted substantia nigra dopaminergic cells, leading to reduced motor control [116]. Interestingly, MM are one of the symptoms that can be present in PD [83]. There is neurophysiological evidence that MM in PD are the result of M1 activation ipsilaterally to the intended movement rather than resulting from the presence of an ipsilateral CS pathway [83]. Hence, it has been hypothesized that MM in PD are the result of a deficiency of the basal ganglia to support the cortical network that is believed to underlie nonmirroring transformations necessary for unilateral movements [49, 83]. Dysfunctional basal ganglia should have a consequence on its output towards the SMA, which is what is seen in PD, where cerebral blood flow in SMA is reduced compared to healthy individuals [117]. Further evidence that the SMA is impaired in PD comes from the fact that the early BP is reduced [118]. An alternative explanation for the presence of MM in PD is that abnormalities of the basal ganglia can lead to a loss of cortical inhibition, which may produce excessive activation in superfluous muscles when performing voluntary movement [119]. Indeed, intracortical inhibition has been shown to be reduced in untreated PD patients, reflecting abnormal excitability of the motor pathway [120].

To perform lateralize unilateral movements, the brain relies on a distributed network which seems to imply the dPMC, the SMA, and the basal ganglia. The disruption of any part of this network enhances the natural tendency towards symmetrical bimanual movement. But only modest effects are seen when disrupting parts of this network suggesting that none of these brains regions are solely responsible for the nonmirroring process.

## 4. MM and Aging

MM are seen in healthy children up to the age of 10 years and probably reflect the fact that a fully matured CC is associated with greater IHI, underlying the ability to perform complex unilateral motor tasks [5]. As was seen earlier, if MM continue after that age they are considered abnormal and are usually the consequence of the presence of an ipsilateral fast-conducting CS tract originating from both M1s [4]. However, even in the absence of an aberrant ipsilateral projection, an intact CC is needed to restrict motor output in the hemisphere contralateral to an intended movement. As such, if the CC is dysfunctional, for instance in schizophrenia [65], motor abnormalities such as increased motor overflow can be seen [67]. Even in healthy subjects with an intact CC, physiological mirroring can be present, especially when performing complex and fatiguing motor tasks [35]. 

With increasing age, motor overflow also appears to increase [43]. This could be linked to the fact that normal aging is associated with numerous morphological changes within the brain, including atrophy of grey and white matter [121]. Neuroimaging studies have shown that in addition to quantitative decreases in white matter, the quality of the remaining WM is compromised in older adults [122]. In otherwise healthy older individuals, there is a decrease in the size and myelination of CC fibres, which is believed to lead to abnormal transcallosal communication. In turn, this would result in increased motor overflow to the hemisphere ipsilateral to the intended movement and ultimately MM. However, recent findings suggest that the naturally occurring reduction of transcallosal pathways is related to a surprising shift in the link between callosal integrity and IHI. Indeed, it was found that older adults with greater callosal tract integrity also displayed a reduction in IHI and a significantly greater interhemispheric facilitation [123]. This is consistent with the HAROLD model proposed by Cabeza [124] in which it is suggested that age-related increases in bilateral activation may be a compensatory mechanism to maintain good functioning. There is evidence that the HAROLD model may generalize to motor function [125]. This would be consistent with the reported age-related amplification of motor overflow in more demanding tasks in the elderly since increasing the attentional demands of a given task is believed to favor recruitment of bilateral areas. This in turn would mean increased activity in the contralateral hemisphere resulting in mirror activity in the ipsilateral, nonactive hand [44]. It is therefore not surprising that normally occurring recruitment of bilateral brain areas in a more demanding task, as well as the consequent motor overflow observed in healthy adults, seems to be enhanced with increasing age. Furthermore, since transcallosal integrity in older adults is associated with lower levels of IHI and a shift towards interhemispheric facilitation, it could partly explain the higher bilateral brain recruitment that is needed for the elderly to maintain good functioning in demanding tasks, but also as a consequence creating increased motor overflow. Taken together, these data suggest that healthy older adults benefit from interhemispheric cooperation between specific brain areas, which is reflected in higher interhemispheric facilitation, lower IHI, and greater overflow to the contralateral motor cortex [123]. This also suggests an adaptive mechanism since greater motor overflow in older adults is associated with increased dexterity [43]. 

## References

[1] Cincotta M, Borgheresi A, Balestrieri F (2004). Involvement of the human dorsal premotor cortex in unimanual motor control: an interference approach using transcranial magnetic stimulation. *Neuroscience Letters*.

[2] Grefkes C, Eickhoff SB, Nowak DA, Dafotakis M, Fink GR (2008). Dynamic intra- and interhemispheric interactions during unilateral and bilateral hand movements assessed with fMRI and DCM. *NeuroImage*.

[3] Swinnen SP, Young DE, Walter CB, Serrien DJ (1991). Control of asymmetrical bimanual movements. *Experimental Brain Research*.

[4] Cincotta M, Ziemann U (2008). Neurophysiology of unimanual motor control and mirror movements. *Clinical Neurophysiology*.

[5] Mayston MJ, Harrison LM, Stephens JA (1999). A neurophysiological study of mirror movements in adults and children. *Annals of Neurology*.

[6] Ziemann U, Hallett M (2001). Hemispheric asymmetry of ipsilateral motor cortex activation during unimanual motor tasks: further evidence for motor dominance. *Clinical Neurophysiology*.

[7] Müller K, Kass-Iliyya F, Reitz M (1997). Ontogeny of ipsilateral corticospinal projections: a developmental study with transcranial magnetic stimulation. *Annals of Neurology*.

[8] Gazzaniga MS (2000). Cerebral specialization and interhemispheric communication. Does the corpus callosum enable the human condition?. *Brain*.

[9] Innocenti GM (1986). Postnatal development of corticocortical connections. *Italian Journal of Neurological Sciences*.

[10] Armatas CA, Summers JJ, Bradshaw JL (1994). Mirror movements in normal adult subjects. *Journal of Clinical and Experimental Neuropsychology*.

[11] Reitz M, Müller K (1998). Differences between “congenital mirror movements” and “associated movements” in normal children: a neurophysiological case study. *Neuroscience Letters*.

[12] Cohen HJ, Taft LT, Mahadeviah MS, Birch HG (1967). Developmental changes in overflow in normal and aberrantly functioning children. *The Journal of Pediatrics*.

[13] Wolff PH, Gunnoe CE, Cohen C (1983). Associated movements as a measure of developmental age. *Developmental Medicine and Child Neurology*.

[14] Giedd JN, Rumsey JM, Castellanos FX (1996). A quantitative MRI study of the corpus callosum in children and adolescents. *Developmental Brain Research*.

[15] Giedd JN, Blumenthal J, Jeffries NO (1999). Development of the human corpus callosum during childhood and adolescence: a longitudinal MRI study. *Progress in Neuro-Psychopharmacology and Biological Psychiatry*.

[16] Allen LS, Richey MF, Chai YM, Gorski RA (1991). Sex differences in the corpus callosum of the living human being. *Journal of Neuroscience*.

[17] Koerte I, Heinen F, Fuchs T (2009). Anisotropy of callosal motor fibers in combination with transcranial magnetic stimulation in the course of motor development. *Investigative Radiology*.

[18] Koerte I, Eftimov L, Laubender RP (2010). Mirror movements in healthy humans across the lifespan: effects of development and ageing. *Developmental Medicine and Child Neurology*.

[19] Leocani L, Cohen LG, Wassermann EM, Ikoma K, Hallett M (2000). Human corticospinal excitability evaluated with transcranial magnetic stimulation during different reaction time paradigms. *Brain*.

[20] Liepert J, Dettmers C, Terborg C, Weiller C (2001). Inhibition of ipsilateral motor cortex during phasic generation of low force. *Clinical Neurophysiology*.

[21] Rossini PM, Zarola F, Stalberg E, Caramia M (1988). Pre-movement facilitation of motor-evoked potentials in man during transcranial stimulation of the central motor pathways. *Brain Research*.

[22] Pascual-Leone A, Valls-Sole J, Wassermann EM, Brasil-Neto J, Cohen LG, Hallett M (1992). Effects of focal transcranial magnetic stimulation on simple reaction time to acoustic, visual and somatosensory stimuli. *Brain*.

[23] Chen R, Yaseen Z, Cohen LG, Hallett M (1998). Time course of corticospinal excitability in reaction time and self- paced movements. *Annals of Neurology*.

[24] Murase N, Duque J, Mazzocchio R, Cohen LG (2004). Influence of interhemispheric interactions on motor function in chronic stroke. *Annals of Neurology*.

[25] Duque J, Mazzocchio R, Dambrosia J, Murase N, Olivier E, Cohen LG (2005). Kinematically specific interhemispheric inhibition operating in the process of generation of a voluntary movement. *Cerebral Cortex*.

[26] Duque J, Murase N, Celnik P (2007). Intermanual differences in movement-related interhemispheric inhibition. *Journal of Cognitive Neuroscience*.

[27] Swinnen SP (2002). Intermanual coordination: from behavioural principles to neural-network interactions. *Nature Reviews*.

[28] Hübers A, Orekhov Y, Ziemann U (2008). Interhemispheric motor inhibition: its role in controlling electromyographic mirror activity. *European Journal of Neuroscience*.

[29] Kobayashi M, Hutchinson S, Théoret H, Schlaug G, Pascual-Leone A (2004). Repetitive TMS of the motor cortex improves ipsilateral sequential simple finger movements. *Neurology*.

[30] Kobayashi M, Théoret H, Pascual-Leone A (2009). Suppression of ipsilateral motor cortex facilitates motor skill learning. *European Journal of Neuroscience*.

[31] Avanzino L, Bove M, Trompetto C, Tacchino A, Ogliastro C, Abbruzzese G (2008). 1-Hz repetitive TMS over ipsilateral motor cortex influences the performance of sequential finger movements of different complexity. *European Journal of Neuroscience*.

[32] Takeuchi N, Chuma T, Matsuo Y, Watanabe I, Ikoma K (2005). Repetitive transcranial magnetic stimulation of contralesional primary motor cortex improves hand function after stroke. *Stroke*.

[33] Giovannelli F, Borgheresi A, Balestrieri F (2009). Modulation of interhemispheric inhibition by volitional motor activity: an ipsilateral silent period study. *Journal of Physiology*.

[34] Ferbert A, Priori A, Rothwell JC, Day BL, Colebatch JG, Marsden CD (1992). Interhemispheric inhibition of the human motor cortex. *Journal of Physiology*.

[35] Meyer BU, Roricht S, Grafin von Einsiedel H, Kruggel F, Weindl A (1995). Inhibitory and excitatory interhemispheric transfers between motor cortical areas in normal human and patients with abonormalities of the corpus callosum. *Brain*.

[36] Heinen F, Glocker FX, Fietzek U, Meyer BU, Lücking CH, Korinthenberg R (1998). Absence of translocal inhibition following focal magnetic stimulation in preschool children. *Annals of Neurology*.

[37] Chen R, Yung D, Li JY (2003). Organization of ipsilateral excitatory and inhibitory pathways in the human motor cortex. *Journal of Neurophysiology*.

[38] Perez MA, Cohen LG (2008). Mechanisms underlying functional changes in the primary motor cortex ipsilateral to an active hand. *Journal of Neuroscience*.

[39] Harris-Love ML, Perez MA, Chen R, Cohen LG (2007). Interhemispheric inhibition in distal and proximal arm representations in the primary motor cortex. *Journal of Neurophysiology*.

[40] Ridding MC, Brouwer B, Nordstrom MA (2000). Reduced interhemispheric inhibition in musicians. *Experimental Brain Research*.

[41] Zijdewind I, Kernell D (2001). Bilateral interactions during contractions of intrinsic hand muscles. *Journal of Neurophysiology*.

[42] Arányi Z, Rösler KM (2002). Effort-induced mirror movements: a study of transcallosal inhibition in humans. *Experimental Brain Research*.

[43] Bodwell JA, Mahurin RK, Waddle S, Price R, Cramer SC (2003). Age and features of movement influence motor overflow. *Journal of the American Geriatrics Society*.

[44] Baliz Y, Armatas C, Farrow M (2005). The influence of attention and age on the occurrence of mirror movements. *Journal of the International Neuropsychological Society*.

[45] Addamo PK, Farrow M, Hoy KE, Bradshaw JL, Georgiou-Karistianis N (2007). The effects of age and attention on motor overflow production—a review. *Brain Research Reviews*.

[46] Hess CW, Mills KR, Murray NMF (1986). Magnetic stimulation of the human brain: facilitation of motor responses by voluntary contraction of ipsilateral and contralateral muscles with additional observations on an amputee. *Neuroscience Letters*.

[47] Zwarts MJ (1992). Central motor conduction in relation to contra- and ipsilateral activation. *Electroencephalography and Clinical Neurophysiology*.

[48] Cincotta M, Giovanelli F, Borgheresi A (2006). Surface electromyography shows increased mirroring in Parkinson’s disease patients without overt mirror movements. *Movement Disorders*.

[49] Giovannelli F, Borgheresi A, Balestrieri F (2006). Role of the right dorsal premotor cortex in “physiological” mirror EMG activity. *Experimental Brain Research*.

[50] Cincotta M, Lori S, Gangemi PF, Barontini F, Ragazzoni A (1996). Hand motor cortex activation in a patient with congenital mirror movements: a study of the silent period following focal transcranial magnetic stimulation. *Electroencephalography and Clinical Neurophysiology*.

[51] Krams M, Quinton R, Mayston MJ (1997). Mirror movements in X-linked Kallmann’s syndrome. II. A PET study. *Brain*.

[52] Cincotta M, Borgheresi A, Boffi P (2002). Bilateral motor cortex output with intended unimanual contraction in congenital mirror movements. *Neurology*.

[53] Bloom JS, Hynd GW (2005). The role of the corpus callosum in interhemispheric transfer of information: excitation or inhibition?. *Neuropsychology Review*.

[54] Lepage JF, Beaulé V, Srour M (2011). Neurophysiological investigation of congenital mirror movements in a patient with agenesis of the corpus callosum. *Brain Stimulation*.

[55] Wahl M, Lauterbach-Soon B, Hattingen E (2007). Human motor corpus callosum: topography, somatotopy, and link between microstructure and function. *Journal of Neuroscience*.

[56] Johansen-Berg H, Della-Maggiore V, Behrens TEJ, Smith SM, Paus T (2007). Integrity of white matter in the corpus callosum correlates with bimanual co-ordination skills. *NeuroImage*.

[57] Bonzano L, Tacchino A, Roccatagliata L, Abbruzzese G, Mancardi GL, Bove M (2008). Callosal contributions to simultaneous bimanual finger movements. *Journal of Neuroscience*.

[58] Fling BW, Benson BL, Seidler RD Transcallosal sensorimotor fiber tract structure-function relationships.

[59] Gould HJ, Cusick CG, Pons TP, Kaas JH (1986). The relationship of corpus callosum connections to electrical stimulation maps of motor, supplementary motor, and the frontal eye fields in owl monkeys. *Journal of Comparative Neurology*.

[60] Leichnetz GR (1986). Afferent and efferent connections of the dorsolateral precentral gyrus (area 4, hand/arm region) in the macaque monkey, with comparisons to area 8. *Journal of Comparative Neurology*.

[61] Meyer BU, Röricht S, Woiciechowsky C (1998). Topography of fibers in the human corpus callosum mediating interhemispheric inhibition between the motor cortices. *Annals of Neurology*.

[62] Andres FG, Mima T, Schulman AE, Dichgans J, Hallett M, Gerloff C (1999). Functional coupling of human cortical sensorimotor areas during bimanual skill acquisition. *Brain*.

[63] Caillé S, Sauerwein HC, Schiavetto A, Villemure JG, Lassonde M (2005). Sensory and motor interhemispheric integration after section of different portions of the anterior corpus callosum in nonepileptic patients. *Neurosurgery*.

[64] Crow TJ (1998). Schizophrenia as a transcallosal misconnection syndrome. *Schizophrenia Research*.

[65] Knochel C, Oertel-Knochel V, Schonmeyer R (2012). Interhemispheric hypoconnectivity in schizophrenia: fiber integrity and volume differences of the corpus callosum in patients and unaffected relatives. *NeuroImage*.

[66] Aydin K, Ucok A, Guler J (2008). Altered metabolic integrity of corpus callosum among individuals at ultra high risk of Schizophrenia and first-episode patients. *Biological Psychiatry*.

[67] Hoy KE, Georgiou-Karistianis N, Farrow M, Fitzgerald PB (2009). Neurological soft signs in schizophrenia: investigating motor overflow. *World Journal of Biological Psychiatry*.

[68] Heinrichs DW, Buchanan RW (1988). Significance and meaning of neurological signs in schizophrenia. *American Journal of Psychiatry*.

[69] Pascual-Leone A, Manoach DS, Birnbaum R, Goff DC (2002). Motor cortical excitability in schizophrenia. *Biological Psychiatry*.

[70] Schott GD, Wyke MA (1981). Congenital mirror movements. *Journal of Neurology, Neurosurgery, and Psychiatry*.

[71] Britton TC, Meyer BU, Benecke R (1991). Central motor pathways in patients with mirror movements. *Journal of Neurology Neurosurgery and Psychiatry*.

[72] Cincotta M, Ragazzoni A, De Scisciolo G, Pinto F, Maurri S, Barontini F (1994). Abnormal projection of corticospinal tracts in a patient with congenital mirror movements. *Neurophysiologie Clinique*.

[73] Ueki Y, Mima T, Oga T (2005). Dominance of ipsilateral corticospinal pathway in congenital mirror movements. *Journal of Neurology, Neurosurgery and Psychiatry*.

[74] Conrad B, Kriebel J, Hetzel WD (1978). Hereditary bimanual synkinesis combined with hypogonadotropic hypogonadism and anosmia in four brothers. *Journal of Neurology*.

[75] Mayston MJ, Harrison LM, Quinton R, Stephens JA, Krams M, Bouloux PMG (1997). Mirror movements in X-linked Kallmann’s syndrome. I. A neurophysiological study. *Brain*.

[76] Farmer SF, Harrison LM, Mayston MJ, Parekh A, James LM, Stephens JA (2004). Abnormal cortex-muscle interactions in subjects with X-linked Kallmann’s syndrome and mirror movements. *Brain*.

[77] Farmer SF, Ingram DA, Stephens JA (1990). Mirror movements studied in a patient with Klippel-Feil syndrome. *Journal of Physiology*.

[78] Royal SA, Tubbs RS, D’Antonio MG, Rauzzino MJ, Oakes WJ (2002). Investigations into the association between cervicomedullary neuroschisis and mirror movements in patients with Klippel-Feil syndrome. *American Journal of Neuroradiology*.

[79] Cincotta M, Borgheresi A, Liotta P (2000). Reorganization of the motor cortex in a patient with congenital hemiparesis and mirror movements. *Neurology*.

[80] Galléa C, Popa T, Billot S, Méneret A, Depienne C, Roze E (2011). Congenital mirror movements: a clue to understanding bimanual motor control. *Journal of Neurology*.

[81] Daskalakis ZJ, Christensen BK, Fitzgerald PB, Roshan L, Chen R (2002). The mechanisms of interhemispheric inhibition in the human motor cortex. *Journal of Physiology*.

[82] Hoy KE, Fitzgerald PB, Bradshaw JL (2004). Motor overflow in schizophrenia. *Psychiatry Research*.

[83] Cincotta M, Borgheresi A, Balestrieri F (2006). Mechanisms underlying mirror movements in Parkinson’s disease: a transcranial magnetic stimulation study. *Movement Disorders*.

[84] Netz J, Lammers T, Hömberg V (1997). Reorganization of motor output in the non-affected hemisphere after stroke. *Brain*.

[85] Oliveri M, Rossini PM, Traversa R (1999). Left frontal transcranial magnetic stimulation reduces contralesional extinction in patients with unilateral right brain damage. *Brain*.

[86] Addamo PK, Farrow M, Hoy KE, Bradshaw JL, Georgiou-Karistianis N (2009). The influence of task characteristics on younger and older adult motor overflow. *Quarterly Journal of Experimental Psychology*.

[87] Cracco RQ, Amassian VE, Maccabee PJ, Cracco JB (1989). Comparison of human transcallosal responses evoked by magnetic coil and electrical stimulation. *Electroencephalography and Clinical Neurophysiology*.

[88] Srour M, Philibert M, Dion MH (2009). Familial congenital mirror movements: report of a large 4-generation family. *Neurology*.

[89] Srour M, Rivière JB, Pham JMT (2010). Mutations in DCC cause congenital mirror movements. *Science*.

[90] Depienne C, Cincotta M, Billot S (2011). A novel DCC mutation and genetic heterogeneity in congenital mirror movements. *Neurology*.

[91] Kullander K, Croll SD, Zimmer M (2001). Ephrin-B3 is the midline barrier that prevents corticospinal tract axons from recrossing, allowing for unilateral motor control. *Genes and Development*.

[92] Finger JH, Bronson RT, Harris B, Johnson K, Przyborski SA, Ackerman SL (2002). The netrin 1 receptors Unc5h3 and Dcc are necessary at multiple choice points for the guidance of corticospinal tract axons. *Journal of Neuroscience*.

[93] Depienne C, Bouteiller D, Méneret A (2012). RAD51 haploinsufficiency causes congenital mirror movements in humans. *American Journal of Human Genetics*.

[94] Gunderson CH, Solitare GB (1968). Mirror movements in patients with the Klippel-Feil syndrome. Neuropathologic observations. *Archives of Neurology*.

[95] Netz J, Ziemann U, Homberg V (1995). Hemispheric asymmetry of transcallosalinhibition in man. *Experimental Brain Research*.

[96] Triggs WJ, Calvanio R, Levine M, Heaton RK, Heilman KM (2000). Predicting hand preference with performance on motor tasks. *Cortex*.

[97] Hammond G, Faulkner D, Byrnes M, Mastaglia F, Thickbroom G (2004). Transcranial magnetic stimulation reveals asymmetrical efficacy of intracortical circuits in primary motor cortex. *Experimental Brain Research*.

[98] Ikoma K, Samii A, Mercuri B, Wassermann EM, Hallett M (1996). Abnormal cortical motor excitability in dystonia. *Neurology*.

[99] Verstynen T, Diedrichsen J, Albert N, Aparicio P, Ivry RB (2005). Ipsilateral motor cortex activity during unimanual hand movements relates to task complexity. *Journal of Neurophysiology*.

[100] Zoghi M, Nordstrom MA (2007). Progressive suppression of intracortical inhibition during graded isometric contraction of a hand muscle is not influenced by hand preference. *Experimental Brain Research*.

[101] Schmied A, Vedel JP, Pagni S (1994). Human spinal lateralization assessed from motoneurone synchronization: dependence on handedness and motor unit type. *Journal of Physiology*.

[102] Duque J, Davare M, Delaunay L (2010). Monitoring coordination during bimanual movements: where is the mastermind?. *Journal of Cognitive Neuroscience*.

[103] Chan JL, Ross ED (1988). Left-handed mirror writing following right anterior cerebral artery infarction: evidence for nonmirror transformation of motor programs by right supplementary motor area. *Neurology*.

[104] Sadato N, Yonekura Y, Waki A, Yamada H, Ishii Y (1997). Role of the supplementary motor area and the right premotor cortex in the coordination of bimanual finger movements. *Journal of Neuroscience*.

[105] Brinkman C (1984). Supplementary motor area of the monkey’s cerebral cortex: short- and long-term deficits after unilateral ablation and the effects of subsequent callosal section. *Journal of Neuroscience*.

[106] Laplane D, Talairach J, Meininger V (1977). Clinical consequences of corticectomies involving the supplementary motor area in man. *Journal of the Neurological Sciences*.

[107] Pandya DN, Vignolo LA (1971). Intra- and interhemispheric projections of the precentral, premotor and arcuate areas in the rhesus monkey. *Brain Research*.

[108] Muakkassa KF, Strick PL (1979). Frontal lobe inputs to primate motor cortex: evidence for four somatotopically organized ’premotor’ areas. *Brain Research*.

[109] Berardelli A, Rothwell JC, Thompson PD, Hallett M (2001). Pathophysiology of bradykinesia in parkinson’s disease. *Brain*.

[110] Shibasaki H, Hallett M (2006). What is the Bereitschaftspotential?. *Clinical Neurophysiology*.

[111] Ikeda A, Luders HO, Burgess RC, Shibasaki H (1992). Movement-related potentials recorded from supplementary motor area and primary motor area. Role of supplementary motor area in voluntary movements. *Brain*.

[112] Cunnington R, Iansek R, Bradshaw JL, Phillips JG (1996). Movement-related potentials associated with movement preparation and motor imagery. *Experimental Brain Research*.

[113] Shibasaki H, Barrett G, Halliday E, Halliday AM (1980). Components of the movement-related cortical potential and their scalp topography. *Electroencephalography and Clinical Neurophysiology*.

[114] Jueptner M, Weiller C (1998). A review of differences between basal ganglia and cerebellar control of movements as revealed by functional imaging studies. *Brain*.

[115] Hoover JE, Strick PL (1993). Multiple output channels in the basal ganglia. *Science*.

[116] Kraft E, Chen AW, Flaherty AW, Blood AJ, Kwong KK, Jenkins BG (2007). The role of the basal ganglia in bimanual coordination. *Brain Research*.

[117] Playford ED, Jenkins IH, Passingham RE, Frackowiak RS, Brooks DJ (1993). Impaired activation of frontal areas during movement in Parkinson’s disease: a PET study. *Advances in neurology*.

[118] Dick JPR, Rothwell JC, Day BL (1989). The Bereitschaftspotential is abnormal in Parkinson’s disease. *Brain*.

[119] Hallett M (2000). Transcranial magnetic stimulation and the human brain. *Nature*.

[120] Ridding MC, Inzelberg R, Rothwell JC (1995). Changes in excitability of motor cortical circuitry patients with Parkinson’s disease. *Annals of Neurology*.

[121] Mattay VS, Fera F, Tessitore A (2002). Neurophysiological correlates of age-related changes in human motor function. *Neurology*.

[122] Seidler RD, Bernard JA, Burutolu TB (2010). Motor control and aging: links to age-related brain structural, functional, and biochemical effects. *Neuroscience and Biobehavioral Reviews*.

[123] Fling BW, Seidler RD (2012). Fundamental differences in callosal structure, neurophysiologic function, and bimanual control in young and older adults. *Cerebral Cortex, EPUB*.

[124] Cabeza R (2002). Hemispheric asymmetry reduction in older adults: the HAROLD model. *Psychology and Aging*.

[125] Calautti C, Serrati C, Baron JC (2001). Effects of age on brain activation during auditory-cued thumb-to-index opposition: a positron emission tomography study. *Stroke*.

